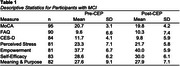# The impact of a multidomain lifestyle and empowerment program on wellbeing in people with MCI

**DOI:** 10.1002/alz70858_103460

**Published:** 2025-12-26

**Authors:** Kayci L. Vickers, Felicia C. Goldstein, Liselotte De Wit, Emily L. Giannotto, Jacquelyn Thelin, Amy D. Rodriguez

**Affiliations:** ^1^ Emory University School of Medicine, Atlanta, GA, USA

## Abstract

**Background:**

Individuals with mild cognitive impairment (MCI) experience cognitive and functional declines that impact their ability to complete everyday activities. These changes negatively impact mood, leading to elevated depression and stress, and reduced feelings of self‐efficacy. Interventions that address psychosocial factors in MCI are critical to support quality of life for those living with cognitive disorders. The present study aimed to evaluate the impact of a multidomain lifestyle program, the Cognitive Empowerment Program (CEP), on wellbeing in people with MCI.

**Method:**

Participants were 95 people with MCI (27.7% female; mean age = 69.1 years, SD = 10.8) referred from Emory's Cognitive Neurology Clinic. Participants completed a 12‐month program comprised of physical, cognitive, and psychosocial interventions. Before and after program completion, participants completed a brief cognitive screener (Montreal Cognitive Assessment; MoCA), and functional status was rated by a knowledgeable informant (Functional Activities Questionnaire; FAQ). Questionnaires were administered to assess depression (Center for Epidemiological Studies‐ Depression; CESD), stress (Perceived Stress Scale; PSS), empowerment (MCI Empowerment Scale; unpublished), and measures of self‐efficacy and meaning and purpose from the NIH Toolbox Emotion Battery. Data were analyzed using descriptive statistics (see Table 1) and paired samples t‐tests.

**Result:**

After completing the CEP program, participants reported significant improvements in stress, t(80) = 2.73, *p* = .01, feelings of empowerment, t[80] = ‐3.01, *p* < .01, self‐efficacy, t(82) = ‐2.11, *p* = .04, and meaning and purpose, t(83) = 2.54, *p* = .01. They did not demonstrate significant change in depression, t(81) = ‐.43; however, depression levels were low pre‐ and post‐program (CESD_Pre_ = 11.7, SD = 4.1; CESD_Post_ = 9.8, SD = 5.9). These findings occurred despite a small decline in cognitive status (MoCA_Pre_ = 20.7, SD = 3.1; MoCA_Post_ = 19.8, SD = 4.2), t(94) = 2.14, *p* = .04, and no change in functional status, t(89) = ‐.93, *p* = .36. Although change in MoCA score is statistically significant, it does not represent clinically meaningful decline.

**Conclusion:**

Results of this study suggest a multidomain lifestyle and empowerment program positively impacts wellbeing and quality of life for those living with MCI. Further research is needed to understand individual factors that impact benefit from comprehensive programs of this nature and whether nonpharmacological lifestyle programs may provide synergistic benefit for pharmacological interventions.